# Defining the Critical Components of Informed Consent for Genetic Testing

**DOI:** 10.3390/jpm11121304

**Published:** 2021-12-05

**Authors:** Kelly E. Ormond, Maia J. Borensztein, Miranda L. G. Hallquist, Adam H. Buchanan, William Andrew Faucett, Holly L. Peay, Maureen E. Smith, Eric P. Tricou, Wendy R. Uhlmann, Karen E. Wain, Curtis R. Coughlin, On behalf of the Clinical Genome CADRe Workgroup

**Affiliations:** 1 Department of Genetics, Stanford University School of Medicine, Stanford, CA 94305, USA; MBorensztein@stanfordchildrens.org (M.J.B.); eptricou@geisinger.edu (E.P.T.); 2 Stanford Center for Biomedical Ethics, Stanford University School of Medicine, Stanford, CA 94305, USA; 3 Genomic Medicine Institute, Geisinger, Danville, PA 17822, USA; mhallquist@geisinger.edu (M.L.G.H.); ahbuchanan@geisinger.edu (A.H.B.); 4 Geisinger Commonwealth School of Medicine, Geisinger, Danville, PA 17822, USA; william.faucett@gm.slc.edu; 5 Genomics, Bioinformatics, and Translational Research Center, RTI Internationa, Research Triangle Park, NC 27709, USA; hpeay@rti.org; 6 Feinberg School of Medicine, Northwestern University, Chicago, IL 60290, USA; m-smith6@northwestern.edu; 7 Department of Internal Medicine, Division of Genetic Medicine, University of Michigan, Ann Arbor, MI 48109, USA; WUhlmann@med.umich.edu; 8 Department of Human Genetics, University of Michigan, Ann Arbor, MI 48109, USA; 9 Center for Bioethics and Social Sciences in Medicine, University of Michigan, Ann Arbor, MI 48109, USA; 10 Autism and Developmental Medicine Institute, Geisinger, Danville, PA 17822, USA; kwain@geisinger.edu; 11 Center for Bioethics and Humanities, Department of Pediatrics, University of Colorado Anschutz Medical Campus, Aurora, CO 80045, USA; CURTIS.COUGHLIN@CUANSCHUTZ.EDU

**Keywords:** genetic testing, genetic counseling, informed consent

## Abstract

Purpose: Informed consent for genetic testing has historically been acquired during pretest genetic counseling, without specific guidance defining which core concepts are required. Methods: The Clinical Genome Resource (ClinGen) Consent and Disclosure Recommendations Workgroup (CADRe) used an expert consensus process to identify the core concepts essential to consent for clinical genetic testing. A literature review identified 77 concepts that are included in informed consent for genetic tests. Twenty-five experts (9 medical geneticists, 8 genetic counselors, and 9 bioethicists) completed two rounds of surveys ranking concepts’ importance to informed consent. Results: The most highly ranked concepts included: (1) genetic testing is voluntary; (2) why is the test recommended and what does it test for?; (3) what results will be returned and to whom?; (4) are there other types of potential results, and what choices exist?; (5) how will the prognosis and management be impacted by results?; (6) what is the potential family impact?; (7) what are the test limitations and next steps?; and (8) potential risk of genetic discrimination and legal protections. Conclusion: Defining the core concepts necessary for informed consent for genetic testing provides a foundation for quality patient care across a variety of healthcare providers and clinical indications.

## 1. Introduction

The principle of informed consent is foundational to the premise that patients and research participants can make autonomous decisions about whether to undergo genetic testing. Critical to this process is that patients and/or research participants must receive and comprehend some specific information in order to make an informed decision and to provide consent. Marteau et al. term this ‘relevant knowledge’ and define it as one of the three components of informed choice [1]. However, various standards exist in determining which information is relevant to provide to the majority of patients. The first, and oldest, was a “medical providers standard”; this is defined by professional guidelines that determine local or national standards of what information to include as part of the informed consent process. The guidelines for Huntington Disease testing were among the first that were endorsed by providers [2] and advocacy organizations (http://hdsa.org/wp-content/uploads/2015/02/HDSA-Gen-Testing-Protocol-for-HD.pdf, accessed on 26 June 2021). While they are focused on the entire genetic counseling process, which includes educational topics and psychosocial assessment and support beyond what may be needed for informed consent, some examples of professional guidelines specific to the genetic testing and counseling process include the National Society of Genetic Counseling (NSGC) Practice Guidelines (e.g., for Alzheimer disease [3], FMR1 [4], and Neurofibromatosis [5]) and the guidelines by the American College of Obstetricians and Gynecologists (ACOG) [6], the American Society of Clinical Oncologists (ASCO) [7], the American Heart Association (AHA) [8], and the Heart Failure Society (HFS) [9].

In contrast to these professional guidelines, the legal case *Canterbury* vs. *Spence* 1972 [10,11] proposed the notion of the “reasonable patient” standard. Driven by patient and family stakeholder views, this approach focuses on the amount and type of information that an average or typical patient would desire and expect to know [12,13,14,15]. Regardless of who defines the relevant content for a typical informed consent conversation, and whether it is a clinical or research setting, there is some general consensus that patients are often overwhelmed by the large volume of educational information that is conveyed and that they frequently find it irrelevant to their questions [16]. This is true both for patients with lower health literacy who may be overwhelmed by the information, and also, in our experience, for patients with high health literacy who may focus on information that is not central to the decision. In response to this, more than two decades ago, a “generic” consent model for carrier testing for reproductive risk information was proposed in order to provide sufficient information without overburdening patients [17,18]. As genetic testing panels have expanded in recent years, additional models have been proposed to help address the ‘information overload’ issue; these include a “tiered and binned” model that was proposed in the context of multiplex cancer susceptibility testing and a similar “tiered-layered-staged” model for personal genome testing [19,20,21,22]. Each of these models tries to find ways to reduce information in general, often by categorizing information either in conceptual ways (e.g., genetic test results can provide information that may vary in terms of the specific risks, including conditions beyond the differential diagnosis, and may be based on context such as personal and family history), or based on categories of potential results (e.g., medically actionable or not; low, medium, or high risk).

Despite this existing literature, there remains no clear agreement either professionally or with patient stakeholders about what concepts are truly critical to allow a patient to meaningfully consent to undergo (or decline) genetic testing. The Consent and Disclosure Recommendations (CADRe) workgroup of the NIH funded Clinical Genome Resource project (clinicalgenome.org) has been focused on proposing and standardizing approaches to communication about genetic testing beyond the traditional genetic counseling model. The CADRe framework proposes that there are many clinical indications where a targeted discussion or brief communication may be an appropriate pretest communication approach [23,24]. The next step in our work, through the present study, is to find consensus on the key elements that encompass the minimum necessary and critical concepts that underlie an informed consent process for genetic testing.

## 2. Materials and Methods

This study used an expert consensus process to define the critical concepts required for informed consent for genetic testing in a clinical setting. We focused on clinical genetic testing primarily because the research on the genetic testing consent process may have additional considerations related to study protocols and IRB requirements. The Stanford University Institutional Review Board (IRB) reviewed this study and declared it exempt.

### 2.1. Survey Methodology

We modeled the consensus study process after the approach of a modified Delphi method [25]. Specifically, our method involved multiple phases. First, a “gathering” phase, when a list of concepts was developed through a literature review. Next, several rounds of surveys were distributed to expert respondents in order to prioritize the concepts, provide feedback on the group’s scoring, and then allow revision of scoring to take into account the anonymous feedback from the group. Finally, analysis determined which concepts have developed consensus within the process.

### 2.2. Literature Review

We completed a literature review to develop a comprehensive list of the concepts involved in the consent process for genetic testing. The review identified publications listed in PubMed before June 2019 using the keywords “informed consent”, “consent”, “consent form”, AND “genetic or genomic” AND “counseling.” We then limited the papers reviewed to those that covered germline genetic testing in clinical and research settings and focused only on those that described the content of either consent forms or of genetic counseling that was part of the informed consent process. We also reviewed the relevant practice guidelines, consent forms from clinical laboratories offering exome testing, and direct-to-consumer genetic testing company websites’ educational materials. The concepts were extracted from the results of the literature review, combined with concepts extracted from prior focus groups with both patients and providers that were conducted by the Clinical Genome project’s CADRe workgroup [23] and discussed by members of the CADRe workgroup. This resulted in a list of 77 concepts across five broad categories: (1) scope of the test (N = 20), (2) description of the test and logistics (N = 28), (3) confidentiality and privacy (N = 13), (4) potential benefits (N = 11), and (5) potential risks (N = 5). While some of the concepts in the original list overlapped with others, we purposely kept the list of concepts detailed and comprehensive in order to determine which nuances of a topic were seen as more or less important to study participants. Importantly, we did not define the concepts; experts were left to interpret them on their own.

### 2.3. Recruitment

In our study we focused on experts (defined below), because they brought content knowledge to the prioritization. We also utilized an anonymous process in order to allow equal voice to each participant, so that the more vocal or more esteemed experts were not given more weight than other participants. A critical feature of any consensus process is clearly defining the expert participant population, in this case, individuals who were considered experts in the topic of informed consent for genetic testing. In this study, experts were defined as genetics clinicians (genetic counselors and medical geneticists) or bioethicists with at least 5 years of experience in obtaining informed consent and results disclosure of genetic testing results, or research experience in the ethical, legal, and social implications (ELSI) of genetic testing who practiced in the United States. Experts were identified through their involvement in one of three areas: (1) ethics-related committees; (2) leadership participation in various clinical special-interest groups within genetics organizations including the National Society of Genetic Counselors (NSGC), the American College of Medical Genetics and Genomics (ACMG), the American Society of Human Genetics (ASHG), and Clinical Genome Resource (ClinGen); (3) genetics special-interest groups within non-genetics organizations (e.g., the American Heart Association and the American Society of Clinical Oncology (ASCO)). Additional ELSI thought leaders in this area were identified as those who had authored multiple publications included in the literature review. The study participants were provided with USD 75 for each completed round of the surveys, for a maximum of USD 150 each.

### 2.4. Methodology

Between November 2019 and February 2020, we conducted two rounds of surveys using Qualtrics online survey administration software; the surveys were linked using a codename created by each participant at the end of Survey 1. We initially selected a two-round approach based on data that suggest that two to three rounds of surveys are adequate to obtain consensus [25].

In Survey 1 (Supplemental Materials Table S1), participants were asked to rate each of the 77 concepts on a 5-point Likert scale with regards to how critical the topic is in obtaining informed consent to undertake a genetic test in a clinical setting. After ranking every item within a category, they then ranked the top five most important items within the category. This was selected as a way to differentiate preferences in a scenario where a significant number of concepts received the highest ratings. After completing the ratings, rankings, and open-ended questions for all five categories, the participants were asked to provide additional comments about the survey as a whole, including identifying any concepts they felt were missing from the original concept list.

Before completing Survey 2, approximately 2 months later, the participants were given their individual results from Survey 1 and a summary of the group results. Participants were asked to rerate all 77 concepts in light of reviewing this data and rated an additional 12 concepts that were suggested by participants during Survey 1. Participants were asked to comment on a list of the 10 most highly rated concepts, considering whether they thought the concepts included “the minimum necessary and critical information to aid an individual in making an informed decision/consent,” and, if not, which concepts they felt were missing or could be removed. They were subsequently shown a list of 18 ‘second-tier’ concepts and asked if they felt that any of these issues warranted inclusion on a core concept list and why. Lastly, participants piloted and provided open-ended comments on four consent scenarios that were intended to be used for a future national survey that would follow up on the results of the present study. Comments from these scenarios were used to inform discussions as the workgroup developed the ‘final concept list’ in this study.

### 2.5. Analysis

In order to define which concepts were most highly rated by the expert participants of Survey 1, we used SPSS version 26.0 (2019, Armonk, New York, NY, USA) to calculate the means, standard deviations, and modes for each concept. In Survey 1, we also tallied the number of times a concept was ranked in the top five within a category. For the “potential risks” category, since there were only five items, we tracked the top three items. The open-ended comments from both surveys were reviewed and discussed by the entire CADRe workgroup in order to determine which concepts were most highly endorsed for inclusion in the subsequent survey.

We created a list of the top concepts from Survey 1 based on the items that scored a mean >4.0 (out of 5.0) and were also frequently ranked in the top-five lists. To ensure that we did not miss any critical concepts with these cutoffs, we created a second-tier of concepts that met one but not both of the criteria above; for example, concepts that either scored between 3.75–3.99 but were frequently ranked in the top five, or concepts that had a score >4.0 but were not frequently ranked in the top five.

To identify the final consensus list of the core concepts that would allow “the minimum necessary and critical information to aid an individual in making an informed decision/consent”, the CADRe workgroup examined the mean responses for two rounds of survey results, using a combination of the criteria described above and looking for consistency across both surveys, as well as the open-ended comments from participants. The workgroup combined some closely related items to avoid repetition. 

## 3. Results

One hundred and thirty-two potentially eligible experts were identified. After excluding 36 individuals who were members of the ClinGen Steering Committee or who were genetics laboratory directors without patient-facing roles, and 8 without publicly available emails, 88 potential participants were emailed in November 2019. Three emails were undeliverable, and 32 confirmed their interest through an eligibility survey (32/85, or a 37.6% initial response rate), one of whom was ineligible based on years of experience. Ultimately, 25 individuals (9 medical geneticists, 8 genetic counselors, and 9 bioethicists) completed Survey 1 (Table 1; 80% of those who expressed initial interest and were eligible), and 23 completed Survey 2, representing a 92% retention rate.

Table 2 shows the levels of agreement for the highest-ranking concepts in each of the two surveys and the closely related concepts that were combined by the workgroup. Supplemental Table S1 shows the mean levels of agreement and rankings for each of the original 77 concepts plus the 12 additional concepts that were ranked in Survey 2.

Thirteen respondents felt that concepts were missing from the concept list presented in Survey 2, with 12 adding open-ended comments; 8 felt that concepts could be removed. The concepts mentioned by more than one participant as missing from the list included: the uncertainty of benefit from genetic testing, general test limitations, how likely it would be for the test to find ‘an answer,’ how data is used and by whom, and issues around insurance and/or discrimination. Concepts mentioned as potentially removable included testing being voluntary, information about management, and the notion that some of the concepts on the list were overlapping.

Sixteen participants (70%) wrote comments regarding the list in general. Ten participants were supportive of the proposed list.

*“I agree that it covers the concepts that most patients want to know about genetic testing.”*

*“I think the list is helpful and focused. It should help improve the efficiency of obtaining consent and will probably improve the understanding of the people getting testing by simplifying the process.”*

*“The list should allow for an informed decision. Some patients/families will have additional needs/interests which can be addressed during time allotted for questions.”*

Two added that they had no further comments, and one highlighted that the survey and their responses “underlines the challenges in streamlining consent”. Two participants highlighted items that they still felt were missing from the proposed concept list: the manner in which the sample will be obtained (and its respective invasiveness) and how potential results will be interpreted.

Finally, when participants reviewed the list of the 18 second-tier concepts, the most highly endorsed potential concepts were: test limitations (12/23; 52%), variant interpretation will change over time (10/23, 44%), and the potential for genetic discrimination or stigma (9/23, 39%). Of note, six people, at least some of whom overlapped with those who endorsed including “genetic discrimination or stigma”, also endorsed “GINA (Genetic Information Nondiscrimination Act) and relevant state laws provide some protection”. Most of the comments regarding GINA/discrimination issues stressed that there are important issues that people may not know about, and may actively wonder or worry about, that should be discussed prior to testing.

*“I think this [GINA] can be wrapped into ‘potential risks for genetic discrimination and stigma, but patients should understand what is/isn’t protected legally before proceeding with testing.”*

### Development of the Final Consensus List

**Figure 1:**
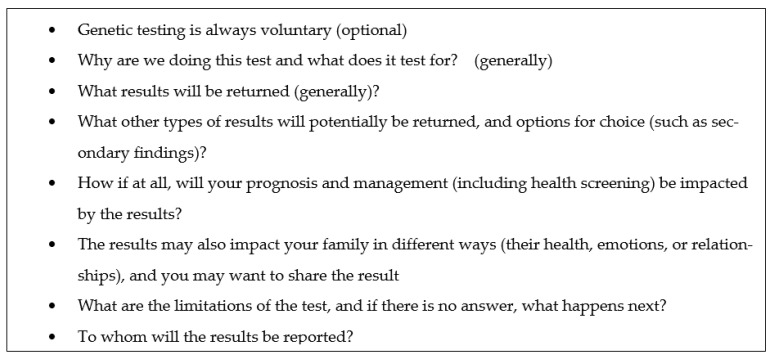
Final list of “necessary and critical” concepts for informed consent for genetic testing.

Eight concepts (Table 2; Figure 1) met the criteria for being highly rated on both surveys (mean scores ≥ 4.0 and being the most highly ranked among the top 5 within each group). One concept met the criteria for mean scores ≥ 4.0 on both surveys but was not among the most highly ranked concepts (“you may learn the cause of the indication for which testing was done”). All of these items demonstrated a ≤ 0.18 change in mean score between the two surveys, demonstrating consistency in views among study participants. Two additional concepts that were added in response to open-ended comments in the first survey had a mean score > 4.0 in Survey 2: “why are we doing the test?” (mean 4.22) and “will there be further testing if no answer (on the genetic test)?” (mean 4.48). Two additional concepts that had been highly ranked in the top 5 in Survey 1 increased their ratings to > 4.0 in Survey 2, such that they met both core criteria for being highly ranked: “we may have information on how to screen/treat some identifiable conditions” (3.89 to 4.16) and “you may learn unexpected information about family relationships” (3.96 to 4.04). One additional concept from the second-tier concepts, “what are the limitations of the test?”, demonstrated a slight increase in mean score (from 3.84 to 3.95) but achieved 52% support for inclusion (n = 12/23) when respondents were specifically queried, and the example scenarios often had unsolicited comments that this topic was missing, so it was retained for inclusion on the final concept list. Finally, given the controversy between our expert participants over whether to include genetic discrimination in the final list, we retained it for further consideration in future studies.

## 4. Discussion

Over the past several years, the CADRe workgroup has taken up the question of how to “operationalize new approaches to provide patients with the education and support that each needs and desires when undergoing genetic testing” [23]. Prior studies of patients suggest that they desire a more straightforward and understandable consent process [16,23,26] with more specific and tailored information provided when test results are disclosed. However, while much discussion and debate has occurred about the concepts to include as part of pretest informed consent around genetic testing, we are not aware of previous studies that operationalize various approaches to informed consent in this concrete way. Our current study developed consensus on a small list of content areas that experts with significant experience obtaining informed consent for genetic testing (geneticists, genetic counselors, and bioethicists who conduct research on informed consent for genetic testing) deemed “necessary and critical” to the process. Having done so, our results suggest that it is feasible to develop a narrow list of concepts that is not tied to any specific condition and that could serve as a ‘starting place’ for clinicians to discuss how best to facilitate informed consent discussions before genetic testing.

This study highlighted the following concepts as critical (Figure 1): the voluntariness of testing; the general reasons why the test is being performed and what is being tested for; the types of results returned; the limitations of testing; to whom the results will be reported; the impact results may have on prognosis, management, and family dynamics; and the potential for discrimination and stigmas. While they are focused on clinical genetic testing, many of these mirror concepts required as part of research consent: voluntariness of testing, the nature and purpose of the test, and the potential benefits and risks (including, in this case, the impact on the self and family, and the potential for discrimination and stigma). This is also highly consistent with our own prior studies that have shown that patients and families preferred to understand what is being tested for and why, what management changes might occur depending on test results, and the insurance implications. Similarly, non-genetics providers also prioritized reasons for testing and the potential impact on management, as well as test limitations and the likelihood of a positive result [23].

We propose that this list is tractable for both patients and clinicians, and provides all clinicians, whether genetics specialists or not, a focal point to begin the discussion about genetic testing with a patient. This concept list does not imply that only these concepts are important in order for a patient to be able to make an informed decision about whether to undergo genetic testing, but rather it prioritizes a ‘most critical’ list as a starting place for discussion, so that clinicians can avoid overwhelming patients with information that is potentially superfluous to their decision-making process. It may well be that some concepts, particularly the ‘second-tier’ concepts, are highly critical for a minority of patients in the pretest consent process, or that they are more uniformly important in the post-test genetic counseling process when the results are available. Furthermore, the core-concept list recognizes that the traditional genetic counseling process may go beyond the concepts required to allow patients to make informed decisions about genetic testing since these sessions often include information regarding the logistics of sample collection and the return of results. Given that our prior work suggests that, for most predictive genetic testing, an abbreviated (‘targeted discussion’) approach would be feasible and appropriate for many testing indications [24], this list would provide guidance to clinicians regarding what concepts might be included in a pretest discussion about genetic testing. Additionally, having a broad framework of consent concepts that is agnostic to the genetic condition or gene for which testing is provided would potentially allow a clinician of any specialty background to feel prepared to identify the patient-specific issues, including clinical testing indication and personal and family history, that would allow tailoring of the content and appropriate triaging of referrals.

We anticipate that there will be some genetics providers who feel uncomfortable with any concept list that attempts to prioritize concepts for informed consent. The recent US-based 21st Century Cures Act Information Blocking Rule (https://www.federalregister.gov/documents/2020/05/01/2020-07419/21st-century-cures-act-interoperability-information-blocking-and-the-onc-health-it-certification, accessed on 26 June 2021), along with laws that allow for the direct release of genetic testing results to patients, may heighten this concern if they lead to limits in post-test counseling, potentially making some providers worry that it is even more critical to provide as much information as possible during the pretest consent conversation. Rather, we would emphasize that tailoring a pretest genetic testing discussion to these points allows a patient either to make an informed decision or to request further information, and that information beyond these points may be best suited to a post-test conversation when providers can contextualize the results and their implications in a focused manner. By shortening the pretest communication process to the discrete set of concepts that this study proposes, we expect that patients will still be able to make informed decisions about genetic testing, providers will feel more comfortable that they are covering the key concepts, and access may be improved through both the broadening pool of providers who are able to facilitate the genetic testing process as well as the shorter time frame required, allowing more patients to be seen in relevant clinics. This hoped-for improvement to access to quality care remains to be tested.

## 5. Limitations

As is the case in most consensus studies, the results of this study provide insight into what ‘experts’ think about a particular topic. Our study characterized experts as US-based genetics care providers (geneticists and genetic counselors) with more than 5 years of experience obtaining consent for genetic testing, as well as bioethics researchers who research and write about the topic. While our demographics suggest that we obtained a range of opinions from people who practice in different clinical areas, different geographic parts of the USA, and with different amounts of experience, it is possible that the views of these experts do not represent the broader professional attitudes (particularly those of non-genetics providers), or even the attitudes that patients and families would have towards the same concepts.

Our methods had several limitations that are worth considering. First, we purposefully started with a long list of potential concepts that were extracted from the literature about both clinical- and research-based genetic testing consents. Both the fact that we had a long and somewhat granular list, and the fact that it included some concepts that were more focused on research genetic testing may have distracted the expert respondents from some concepts that may otherwise have ‘risen to the top’ as highly important. Given that we did not provide specific definitions for each concept, it is also possible that our experts interpreted concepts (for example “test limitations”) in different ways. Finally, we opted to take the final list of concepts back to the CADRe workgroup for a consensus discussion after two rounds of the survey process. It is possible that our determinations would have been different had we held the final consensus discussions with the experts who engaged in the survey process.

## 6. Conclusions

Our study showed that it is possible to reach expert consensus on a minimal list of concepts that are critical to facilitate informed consent in patients considering genetic testing, and we anticipate the medical genetics community will robustly debate whether these concepts are the ‘right’ concepts. Future research directions could include considering whether this concept list could also be applied to somatic genetic testing, pharmacogenomic testing, and direct-to-consumer testing, and also if it could be applied in a research genetic testing context.

Our next steps include determining if a broader sample of medical geneticists and genetic counselors agree that these concepts would allow patients to make informed decisions in a variety of clinical scenarios and indications, and assessing whether these views of critical concepts are supported by patients and families who have previously undergone genetic testing across a broad range of conditions.

## Figures and Tables

**Table 1:** Demographics of participants (N = 25).

**Professional affiliation**	N (%)	
Medical geneticists	9 (36%)	
Genetic counselor	8 (32%)	
Bioethics/researcher	9 (32%)	
**Gender**		
Male	8 (32%)	
Female	15 (60%)	
Prefer not to say	2 (8%)	
**Age**		
25–34	3 (12%)	
35–44	7 (28%)	
45–54	9 (35%)	
55–64	6 (24%)	
**Region of US**		
Northeast	3 (12%)	
Mid-Atlantic	7 (28%)	
South	1 (4%)	
Midwest	5 (20%)	
Mountain	5 (20%)	
West	4 (16%)	
**Clinical Settings**		
	Now *	Past for >1 year *
Cancer genetics	9 (36%)	9 (36%)
Prenatal/reprogenetics	6 (24%)	5 (20%)
Pediatrics/general medical genetics	9 (36%)	10 (40%)
Cardiogenetics	5 (20%)	7 (28%)
Neurogenetics	6 (24%)	7 (28%)
Other (adult genetics, immunology, DSD, precision medicine, internal med, palliative med)	4 (16%)	4 (16%)
**Experience with consent for genetic testing**		
Regularly	15 (60%)	
Occasionally	2 (8%)	
Not currently but 5+ years over career	3 (12%)	
Never/less than 5 years over career	5 (20%)	

* Responses add to >100% as participants could select multiple options.

**Table 2:** Most highly ranked concepts from the consensus process.

Concept	Survey 1 N = 25 Mean ± SD	Survey 2 N = 23 Mean ± SD	Comments
What is the condition we are testing for? You may learn the cause of the indication we are testing for. Why are we doing the test?	4.76 ± 0.523 4.12 ± 0.881 Added in comments	4.91 ± 0.288 4.30 ±.765 4.22 ± 2.07	Combined in final list as “Why are we doing this test and what does it look for?”
What results will be returned (generally)?	4.68 ± 0.557	4.70 ± 0.559	
How, if at all, will management be impacted by the results? There may be an impact on your personal health through a diagnosis. We may have information on how to screen/treat some identifiable conditions.	4.40 ± 0.707 4.36 ± 0.810 4.16 ± 0.850	4.43 ± 0.590 4.50 ± 0.514 3.89 ± 0.583	Combined in final list as “How, if at all, will your prognosis and management (including health screening) be impacted by the results?”
Genetic testing is always voluntary (optional). You have the right not to know about your genetic status.	4.20 ± 0.707 4.04 ± 0.978	4.22 ± 0.671 3.91 ± 0.996	Combined in final list as “Genetic testing is always voluntary”
A diagnosis may also impact your family in different ways (their health, emotions, or relationships). You may learn unexpected information about family relationships. How to share with family.	4.12 ± 0.927 4.04 ± 0.790 Added in comments	4.00 ± 0.686 (broadly), 3.96 ±.706 3.96 ± 1.107	Combined in final list as “The results may also impact your family in different ways (their health, emotions, or relationships), and you may want to share the results.”
What other types of results will potentially be returned, and options for choice (such as secondary findings)?	4.04 ± 0.790	4.17 ± 0.650	
To whom will the results be reported?	4.00 ± 0.816	4.00 ± 0.739	
What are the limitations of the test? Will there be further testing if no answer?	3.84 ± 0.800 Added in comments	3.96 ± 0.767 4.48 ± 0.805	Represented in top 5 on Survey 1; 52% (12/23) support adding in comments on S2
There is a potential risk for genetic discrimination. There may be risks for discrimination or stigma (insurance, etc.). GINA and relevant state laws provide some protection.	3.08 ± 0.997 3.91 ± 0.949 3.76 ± 0.926	3.52 ± 0.790 3.61 ± 0.778 3.87 ± 0.757	All three items highly ranked in top 5 on S1. Of participants, 43% (9/21) supported adding at least one of the items in comments on S2. Items combined and added to final list as “There is a potential risk for genetic discrimination and/or stigma/GINA and relevant state laws provide some protection.”

## Data Availability

The entire set of summarized data from both surveys is available in the Supplemental Table S1.

## Supplementary Materials

The following are available online at https://www.mdpi.com/article/10.3390/jpm11121304/s1, Table S1. Full results from Survey: Means and Rankings (77 Original Concepts + 12 Added Concepts).
